# A Novel Magnetic Stimulator Increases Experimental Pain Tolerance in Healthy Volunteers - A Double-Blind Sham-Controlled Crossover Study

**DOI:** 10.1371/journal.pone.0061926

**Published:** 2013-04-19

**Authors:** Rudie Kortekaas, Lotte E. van Nierop, Veroni G. Baas, Karl-Heinz Konopka, Marten Harbers, Johannes H. van der Hoeven, Marten van Wijhe, André Aleman, Natasha M. Maurits

**Affiliations:** 1 Department of Neuroscience, University Medical Center Groningen, University of Groningen, Groningen, The Netherlands; 2 Department of Anesthesiology, University Medical Center Groningen, University of Groningen, Groningen, The Netherlands; 3 Department of Neurology, University Medical Center Groningen, University of Groningen, Groningen, The Netherlands; 4 Department of Psychiatry, University Medical Center Groningen, University of Groningen, Groningen, The Netherlands; The James Cook University Hospital, United Kingdom

## Abstract

The ‘complex neural pulse’^TM^ (CNP) is a neuromodulation protocol employing weak pulsed electromagnetic fields (PEMF). A pioneering paper reported an analgesic effect in healthy humans after 30 minutes of CNP-stimulation using three nested whole head coils. We aimed to devise and validate a stimulator with a novel design entailing a multitude of small coils at known anatomical positions on a head cap, to improve applicability. The main hypothesis was that CNP delivery with this novel device would also increase heat pain thresholds. Twenty healthy volunteers were enrolled in this double-blind, sham-controlled, crossover study. Thirty minutes of PEMF (CNP) or sham was applied to the head. After one week the other treatment was given. Before and after each treatment, primary and secondary outcomes were measured. Primary outcome was heat pain threshold (HPT) measured with thermal quantitative sensory testing. Other outcomes were warmth detection threshold, and aspects of cognition, emotion and motor performance. As hypothesized heat pain threshold was significantly increased after the PEMF stimulation. All other outcomes were unaltered by the PEMF but there was a trend level reduction of cognitive performance after PEMF stimulation as measured by the digit-symbol substitution task. Results from this pilot study suggest that our device is able to stimulate the brain and to modulate its function. This is in agreement with previous studies that used similar magnetic field strengths to stimulate the brain. Specifically, pain control may be achieved with PEMF and for this analgesic effect, coil design does not appear to play a dominant role. In addition, the flexible configuration with small coils on a head cap improves clinical applicability.

**Trial Registration:**

Dutch Cochrane Centre NTR1093

## Introduction

Magnetic stimulation of the brain is a safe and non-invasive way to modulate brain function. The best known method is transcranial magnetic stimulation (TMS) and uses strong (1–2 T) and short (<1 ms) pulses. In 1985 Barker *et al*. described the induction of involuntary movement by stimulation of the motor cortex which indicates that TMS is able to induce action potentials in the brain [1].

Weaker magnetic stimulation can be achieved with small or large coils, either commercially available or custom built. This technique often uses pulsed stimulation and is then referred to as pulsed electromagnetic field (PEMF) stimulation. Recently, some evidence has been found that exposure to MRI systems may change mood [2], brain metabolism [3] and brain activation [4]. In humans PEMF appears to have both analgesic [5] and antidepressant [6] effects.

Often commercial stimulation systems (e.g. NeoSync, Inc., PEMF Systems, Inc., CNP Therapeutics Inc.) use a limited number of small coils while in research the use of one or more Helmholz coils is often reported [5]. Helmholz coils are large coils that fit around the head and generate a relatively homogeneous magnetic field. The disadvantage of few coils as well as of very large coils is that it is difficult to investigate which stimulation sites in the brain are most effective for inducing a specific effect. Large coils are also impractical due to their size and weight which impedes their wide spread therapeutic use in either a clinical or even an extramural i.e. domestic setting. By far the most practical stimulation system for wide spread clinical and extramural use would be a small magnetic stimulator that is easy to use and able to selectively stimulate specific brain areas. It should also be able to generate any type of magnetic wave to maximize applicability. The use of multiple light coils on a head cap eliminates subject motion relative to the coils and the coils may be positioned over known anatomical sites. We selected the 10/20 system as used in EEG for the positioning of the coils because this allows for easy coupling of newly found knowledge to EEG findings and to functional neuroanatomy because the brain structures under the electromagnets are known [7]. One of our aims was the construction of such a stimulation system.

The complex neural pulse (CNP^TM^) [8] has been used as a PEMF at low field strength and it was shown to have analgesic efficacy in snails [8], rats [9] and humans [5]. A recent study in humans applied PEMF with the gradient coil of an MR scanner and found a negative correlation between field strength and brain activation in a network of brain areas that respond to pain [10]. A second aim of the present study was to investigate the analgesic potency of the CNP when administered with our own device.

The underlying mechanism of PEMF induced analgesia is poorly understood. There is some evidence for endogenous opioid mediation of PEMF analgesia in animals[8], [11], but in humans the mechanism is largely unknown. It is known that mood has a strong influence on pain experience [12] and improved mood is thus a potential mediator of PEMF analgesia, especially because PEMF was reported to improve mood in depressed patients [6].

Further, dopaminergic tone is correlated to mood and also sensitive to PEMF and TMS [13], [14]. In addition, dopamine has a modulatory effect on pain (for review see [15]) making dopaminergic tone another, although related, possible mediator of PEMF analgesia. Non-invasive assessment of dopaminergic tone in humans requires PET or SPECT imaging. However, there are behavioral markers that are safer, quicker, cheaper and more practical: the speed of finger tapping and the size of handwriting are both highly significantly correlated with central dopaminergic tone as established by PET or SPECT[16], [17]. Our final aim was to find evidence for mediation of an analgesic effect by mood or dopamine release.

We applied the CNP [8] and studied its effects on the experience of experimentally induced heat pain in healthy volunteers. We also assessed emotional state as a potential mediating factor of analgesia and aspects of motor and cognitive performance.

## Materials and Methods

### Ethics Statement

This research has been approved by the Medical Ethical Committee of the University Medical Center Groningen. Informed consent was obtained from the subjects and the clinical investigation was conducted according to the principles expressed in the Declaration of Helsinki.

### Wave synthesis

**Figure 1:**
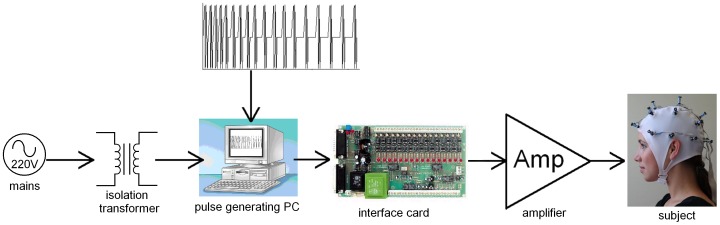
Schematic overview of the hardware. The interface card translates digital values into voltages. The amplifier in turn increases power to generate pulsed magnetic fields in nineteen small electromagnets radially attached to the head cap. Photo by S. Martens, consent to publication was obtained from the subject.

A personal computer (Pentium) and interface card (K8000, Velleman, Gavere, Belgium) were used as Arbitrary Waveform Generator (see figure 1). Digitization resolution was 3 ms, no extra filtering was applied.

The computer ran a bash shell on Debian Linux (www.debian.org). C++ programs were written that contained instructions for wave generation using the libk8000 library (freshmeat.net/projects/libk8000). Compilation with g++ resulted in one small (<100 kB) executable for each wave.

### Wave amplification

To increase the low power output of the K8000 a DC coupled amplifier was built (figure S1). The amplifier had a medical power supply and an isolation unit as additional safety features.

### Magnetic field generation and head cap

The electromagnets consisted of 25 mm long, 9 mm thick reed relays (Reed Relay 275–232, Radio Shack, Fort Worth, TX, USA) of which the reed switch was replaced [18] by an M2×30 mm grade 2 steel bolt, transforming them into iron core electromagnets. Measurements of electrical properties with an RLC bridge on a single coil yielded the following values: resistance: 245 Ω, inductance at 100 Hz: 122 mH, at 1 kHz: 89 mH (without iron core: 13.5 mH).

Nineteen of these electromagnets were radially attached to a regular EEG cap with a chin strap (SU-60 and KR, MedCaT, Erica, The Netherlands) using non-metallic nuts on the inside of the cap. Electromagnets were positioned according to the international 10/20 system for EEG electrodes (figure 1).

#### Safety

All electrical equipment was powered through a medical isolation transformer (H01.96.00, Jansen Medicars, Maarssen, Netherlands) (see figure 1).

The entire setup was tested with an International Safety Analyzer (601PRO, BIO-TEK Instruments Inc., Winooski, VT, USA) as a class I, type B device according to norm 601 of the International Electrotechnical Commission (IEC; 1988). Leak currents to earth were below 20% of the norm, patient leak currents were below 1.8% of the norm (always below 10 µA) at a current consumption of 0.2 A. The device passed all tests for a class I type B clinical device.

#### Characterization of the setup

**Figure 2:**
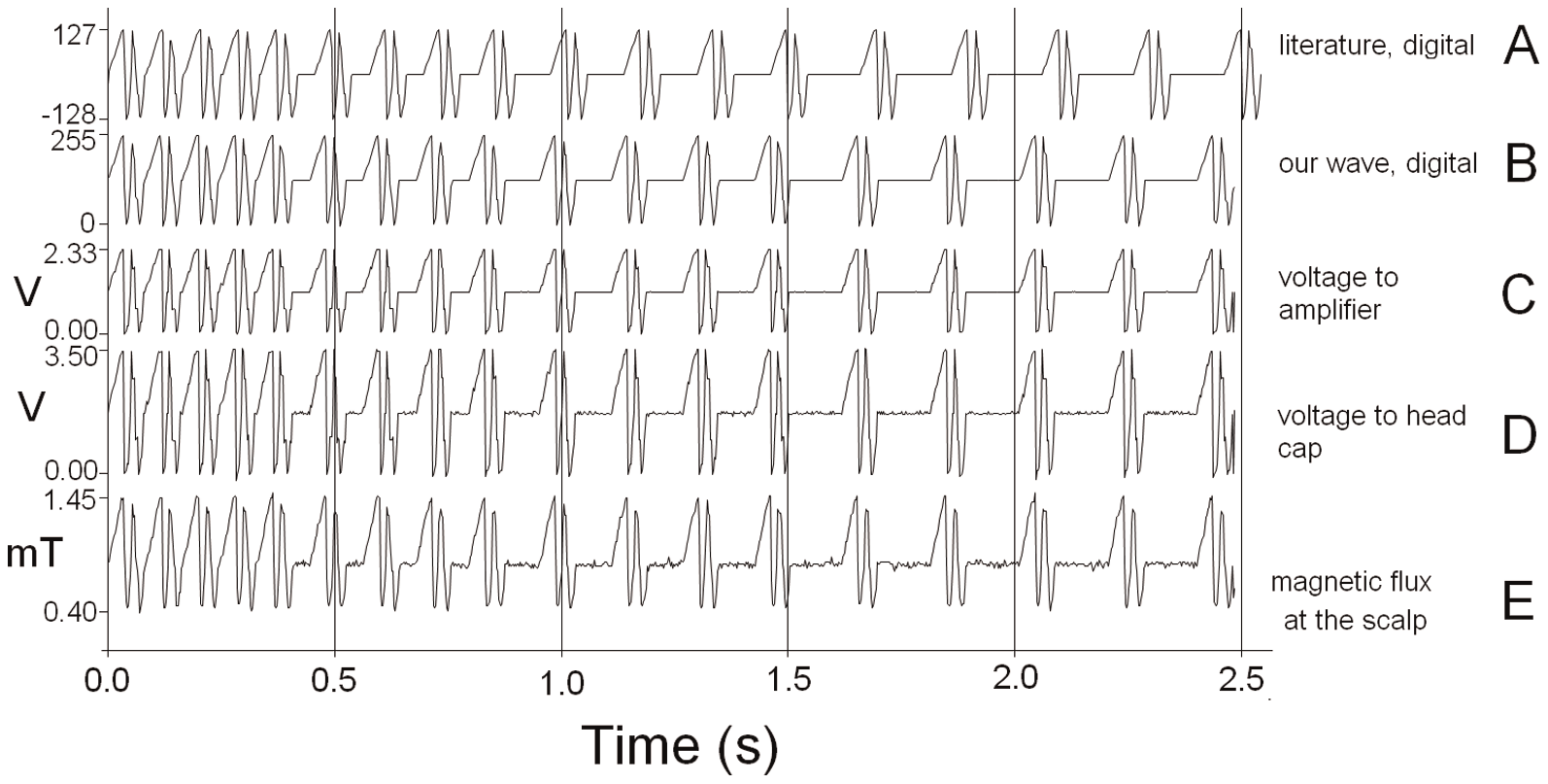
Different stages of the CNP signal. The digital version of the CNP wave as published in the literature[22] (A) and used here (B), was converted by PC and interface card to the analogue version (C), which was amplified (D) and converted to a magnetic wave (E), all to the same time scale.

Maximum magnetic flux density was 1.45 mT at each electromagnet (see figure 2).

#### Frequency response

DC shifted sine waves (min 0 V, max +3.47 V) of different frequencies were generated with a Function Generator (Model 110, Wavetek, San Diego, CA, USA) and a DC power supply and were then used as input to the amplifier.

The following were measured: voltage into the amplifier, voltage out of the amplifier and magnetic flux density (Gauss-/Teslameter, FH 54, with an axial Hall probe, HS-AGB5-4805, Magnet-Physik, Cologne, Germany) at the scalp side of one of the coils.

**Figure 3:**
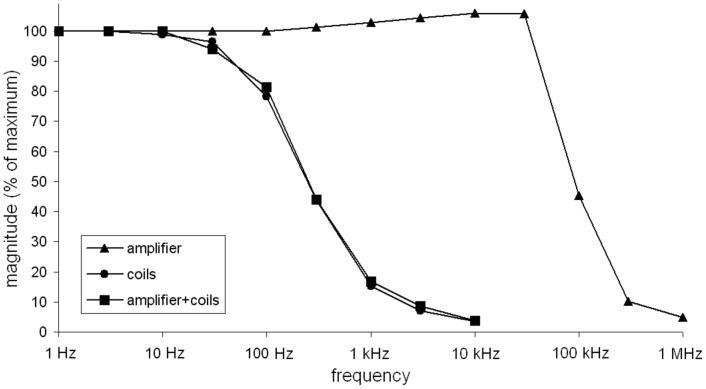
Frequency characteristics of amplifier, coils and amplifier + coils.

The coils acted as a low pass filter, limiting the frequency response of the system: while the amplifier had its 50% frequency around 90 kHz, the coils showed a 50% frequency at about 300 Hz (figure 3).

#### Legal and ethical

Before clinical testing of the stimulator, a description of the equipment and a copy of the Insurance Certificate were filed with The Dutch Health Care Inspectorate (Inspectie voor de Gezondheidszorg) to comply with legislation.

The study conformed to national legislation on medical research and was approved by the Medical Ethical Committee of the University Medical Center Groningen, the Netherlands. In addition the study was registered in the Dutch Trial Register (Dutch Cochrane Centre, NTR1093, http://www.trialregister.nl/trialreg/admin/rctview.asp?TC=1093).

Also, we conformed to the Dutch Personal Data Protection Act (“Wet Bescherming Persoonsgegevens” of 2001). Subjects gave written informed consent and received neither financial nor curricular incentives for their participation.

#### Subjects

Twenty healthy volunteers, all native Dutch speakers, were recruited through advertisements on bulletin boards of the University of Groningen. Inclusion criteria were: 18–80 years old, subjectively healthy. Exclusion criteria were: neurological (e.g. epilepsy) history, psychiatric history, recent use (within four weeks) of prescription or non-prescription psychopharmaca, use of >10 units of coffee per day, use of >10 units of alcohol per day, presence in the body of MRI incompatible implants.

#### Design

This was a single center, double-blind, sham-controlled crossover study conducted in the Netherlands. The within-subjects design was balanced for treatment order. All subjects received a sham and an active treatment at the same time of the day with one week in between. By using a random number generator on the stimulus PC, neither the subject nor the experimenter was aware of the nature of the treatment. The code was broken after all twenty subjects had been treated twice. Also, two field strengths were tested in order to investigate dose (field strength) effects on the outcome parameters: HIGH (amplitude 1.1 mT) and LOW (amplitude 0.4 mT). Half of the subjects received HIGH and half received LOW as their active treatment.

#### Intervention

During a session the volunteers were seated behind a desk while wearing the treatment cap. Each session consisted of four blocks of fifteen minutes each. The blocks were identical in all aspects, except that during the first and last block only zeroes were sent to the DAC. During the second and third block either PEMF (LOW or HIGH field, one option per subject) or sham (all subjects) was applied through all electromagnets so that a total of 30 minutes of PEMF or sham stimulation was applied in each session. For sham too, only zeroes were sent to the DAC.

The applied field was measured afterwards with a tesla meter (FH 54, Magnet-Physik, Cologne, Germany) and a digital storage oscilloscope (DSO-101, Syscomp Electronic Design Limited, Toronto, Canada) and is presented in figure 2. The used PEMF is based on a published pattern [19], but for simplicity we removed all trailing zeroes except one and shifted it to positive only. This does not have a direct influence on the first time derivative of the field strength, which is proportional to the induced current according to Faraday's law. Every integer was converted to a voltage and presented for approximately 3 ms, resulting in a wave duration of just under 2.5 s. For the LOW treatment all numbers in the digital wave were divided by two (the relationship between this digit and the resulting field strength is not linear). The sham treatment had the same length and time resolution, but all points on the digital wave had the value of 0.

#### End points–general

Several tests were selected to sample the emotional, sensory, motor and cognitive domain. Two emotional inventories were administered before and after all other tests. The other tests were performed in four consecutive identical blocks of fifteen minutes each. For all parameters, the initial value was subtracted from the other values on a per subject per session basis. In case the measure was repeated within a block, the mean value per block was calculated.

#### Thermal QST

Warmth detection threshold (WDT) and heat pain threshold (HPT) were taken as indicators of sensory and pain perception, respectively. WDT and HPT were measured with thermal quantitative sensory testing (tQST) with a computer controlled thermode (Thermotest, Somedic, Hörby, Sweden). The thermode was held in the non-dominant hand with the heatable surface at a thenar palmar position.

**Figure 4:**
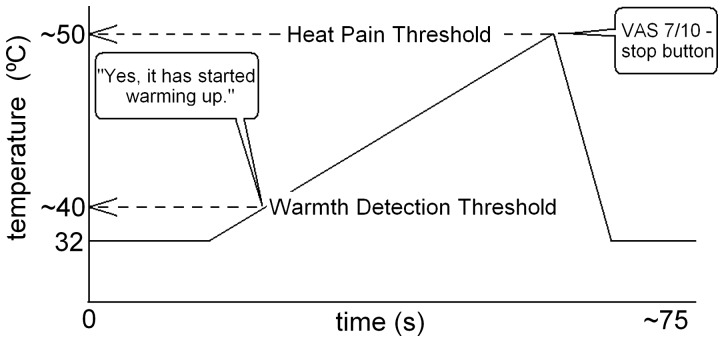
Schematic of thermal quantitative sensory testing (tQST) method for assessing Warmth Detection Threshold (WDT) and Heat Pain Threshold (HPT). See text for details.

The thermode temperature started at 32°C and started warming up at 0.3°C/s at an unpredictable moment (figure 4). Subjects were instructed to report the moment at which they noticed that the thermode had started to warm up. The temperature at which this happens is called the WDT.

When the temperature of the thermode induced a pain sensation with an intensity of 7 on a scale from 0 (no pain) to 10 (severe pain) subjects pressed an 'escape button' which resulted in immediate and rapid cooling (3°C/sec) of the thermode to 32°C. The temperature at which they pressed the button is called the HPT and this variable was the primary outcome.

WDTs and HPTs were always measured *in triplo* and two of these triplets were measured during each 15-minute time block. For each triplet, the median was considered for analysis.

### Finger Tapping

The speed of finger tapping was measured with a hand counter (‘hand tally’). Subjects were instructed to hold the counter in the dominant hand and to press the button with the thumb of the same hand as often as possible in 20 s. During each 15-minute block this was measured twice *in duplo*. For each doublet, the average was considered for analysis.

### Hand Writing

The size of handwriting was assessed by the request to copy a text (single sentence of 24 words, 132 characters) by hand onto a blank piece of paper. This was done once in each 15-minute block, resulting in four time points per subjects per session. The total surface area of the written text (cm^2^) was determined and used for further analysis.

#### Cognition

Once in each 15-minute block Digit-Symbol Substitution Test (DSST) of the Wechsler Adult Intelligence Scale (WAIS)[20] was applied. The DSTT provides a composite measure of attention, working memory, psychomotor speed, processing speed, high-speed visuomotor speed and visuospatial speed. Subjects were instructed to correctly substitute as many symbols by digits as possible in 90 seconds. The number of correct substitutions was used for further analysis.

#### Emotion

To assess emotional state during the experiment, the Dutch versions of the Positive and Negative Affect Schedule (PANAS)[21] and the Profile Of Mood States (POMS) were completed at the beginning and at the end of both experimental sessions. An extra item “happy” was added to the PANAS, resulting in a total of 21 items; they were combined to result in a score for positive associations and a score for negative associations. The POMS resulted in scores on the subscales *Depression and dejection*, *Anger and hostility*, *Fatigue and inertia*, *Vigor and activity* and *Tension and anxiety*.

#### Exit interview

After all measurements subjects were debriefed and asked to report any unusual sensations, moods or thoughts during the experiment. Also they were asked whether they noticed the treatment with magnetic fields.

#### Statistics

For all outcomes the pre-treatment value was subtracted on an individual basis. The primary outcome was then tested across treatments with a one-sided paired t test on the difference scores. Significance was accepted at 0.05. For the secondary outcomes an exploratory analysis was done on the treatments using two-sided paired t tests on the difference scores. In this case, a conservative multiple comparisons correction (Bonferroni, 11 comparisons) led to significance being accepted at 0.0045.

#### Visual presentation

Figures in the results section show the change after treatment relative to the first measurement in the same subject in the same session. Bars indicate means and standard error of the mean.

## Results

Table 1 shows demographic and clinical characteristics for each treatment group. The median (interquartile range-IQR) of weekly coffee consumption was 3 (16.25) units. The median (IQR) of weekly alcohol consumption was 3.5 (4.1) units.

**Table 1:** Demographic characteristics for each treatment group.

Group	n	female (%)	right-handed (%)	mean age (min, max, stdev)
HIGH-sham	5	80	60	29.4 (24, 44, 8.35)
LOW-sham	5	80	80	25.8 (20, 40, 8.07)
sham-HIGH	5	100	80	24.6 (23, 29, 2.51)
sham-LOW	5	100	100	24.4 (22, 28, 2.61)

### Safety, exit interview and blindness

None of the volunteers reported adverse events or other complaints. At the exit interview they were invited to guess which treatment they had just received. There was no relationship between the actual treatment and the subjects' guess.

### Dose-response relationship

There were no statistical differences between data from the LOW and the HIGH treatment, therefore the two treatment groups were combined into one.

### Treatment effects on tQST, motor function and emotion

**Figure 5:**
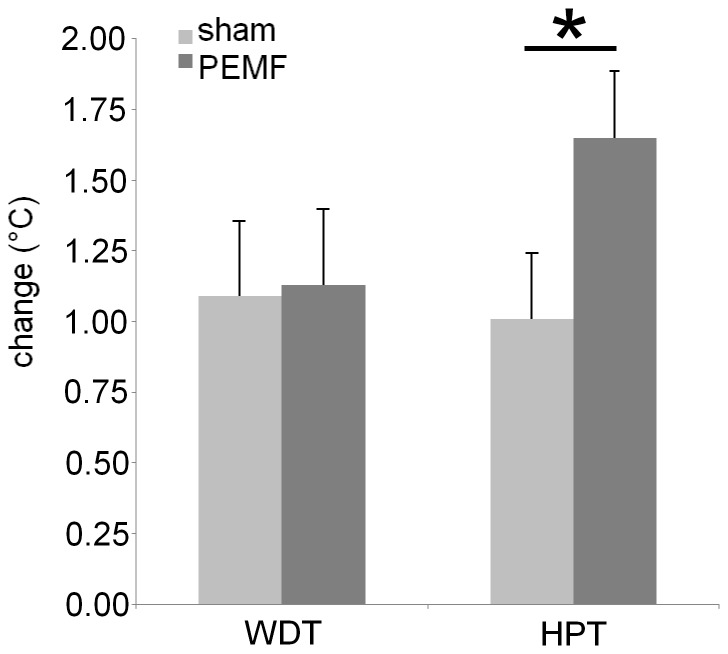
Effects of PEMF treatment on changes in skin sensitivity. Warmth detection threshold (WDT) was unaltered by PEMF (p = 0.455) but heat pain threshold (HPT) increased more after PEMF than after sham stimulation. * significantly different at p<0.05.

Both WDT and HPT increased over the experiment. The primary outcome parameter, HPT was increased more after PEMF than after sham (t(19) = 1.98, p = 0.0313, Cohen's d = 0.613), but for WDT there was no significant treatment effect (t(19) = 0.114, p = 0.455) (figure 5).

For the WAIS symbol to digit substitution task there was a non-significant effect of treatment (t(19) = 2.82, p = 0.0110, alpha crit = 0.0045) with lower performance after PEMF.

The two motor variables, used as indices of dopaminergic function, were both unaltered by the PEMF: finger tapping (t(19) = 0.920, p = 0.369) and handwriting (t(19) = 1.20, p = 0.245).

From the emotional variables the PANAS showed that the change in positive associations was negative i.e. subjects were less positive after the treatment. Likewise, the change in negative associations was positive indicating that subjects were more negative after the experiment. Despite these changes there was no evidence of a significant treatment effect: *positive associations* (t(19) = 0.421, p = 0.679), *negative associations* (t(19) = 0.0785, p = 0.938). Also for the POMS there was no evidence of a significant treatment effect on any of the emotional subscales (all t(19)< = 0.157, all p> = 0.358).

## Discussion

We aimed to construct a novel device for cerebral PEMF stimulation and tested the hypothesis that the stimulation exerted an analgesic effect when applying a wave pattern known as CNP [8].

As hypothesized the weak field PEMF treatment for 30 minutes increased HPT compared to sham stimulation. The effect size as indicated by Cohen's d is 'medium' to 'large'. During sham exposure HPT increased by approximately 1°C. In addition, the PEMF effect added approximately 0.7°C to the HPT. Thus the HPT increasing effects of PEMF were similar in magnitude to the habituation effect that developed over the course of the experiment. Taking into consideration that the thermode temperature increased by 0.3°C/s, treated subjects allowed the already hot thermode to warm up for an additional 2 to 3 seconds on average.

Our PEMF effects on tQST results are in agreement with a previous study [5] which used a very similar time varying magnetic pulse. Despite a significant number of methodological differences (within-subjects vs. between-subjects, nineteen radial electromagnets vs. three orthogonal Helmholz coils) they found a similar result to ours, being an increased HPT due to PEMF treatment and no treatment effect on WDT. They also observed that PEMF effects were more pronounced in women which is compatible with the fact that our effect was robust and that our population was mainly (90%) female. The increased HPT also confirms that the current arrangement of coils, using the international coordinate system derived from EEG, is effective for the induction of experimental analgesia. The trailing zeros in the CNP as described in the patent [22] do not seem to be necessary for its analgesic effect because we omitted them in this study.

This study shows that the PEMF effect appeared to be quite specific for HPT. PEMF treatment had no effect on WDT, indicating that the ability to detect warmth, a non-noxious thermal stimulus, remained unaltered by the pulsating magnetic field. This is in agreement with the literature [5] and it is a very advantageous property for an analgesic treatment: to only reduce pain without reducing a person's sensitivity. Finger tapping speed, handwriting, PANAS and POMS results were all unaffected by the PEMF treatment.

The digit to symbol substitution task showed a non-significant treatment effect with worse performance after PEMF than after sham. The fact that this did not reach significance was because we did not have a hypothesis about cognition so this is a result from an explorative analysis with a conservative multiple comparisons correction. However, there is some biological plausibility because it was recently shown that cognitive control and sensory processing can both be influenced simultaneously by one intervention or manipulation [23]. These considerations combined with the need for analgesic treatments without cognitive side effects, motivate the future study of cognitive performance after PEMF treatment.

In order to gather information concerning the working mechanism of the induced analgesia, we also measured two emotional parameters and two motor parameters that are sensitive to dopaminergic tone. No treatment effects on the emotional state were found so we have no evidence that emotional changes were mediating the PEMF effects on HPT. We also found no treatment effects on the two behavioral indices of dopaminergic tone. Taken together these findings suggest that PEMF analgesia is not mediated by changes in emotion or in central dopaminergic tone.

The fact that pain tolerance was increased does not identify a single neuroanatomical structure as the mediating location because the level of pain tolerance is the end result of the total function of the anterolateral somatosensory system: nociceptors, thin fibers, dorsal horn, ventral commissure, spinothalamic tract, periaqueductal grey and reticular formation, ventromedial, mediodorsal and intrathalamic thalamus, insula and anterior cingulate cortex. The latter two are involved in the emotional aspects of pain such as tolerability and suffering. Therefore, these are plausible areas for mediation of increased pain tolerance and in fact a relatively recent study found support for the notion that brain activation in insula and anterior cingulate cortex as measured with fMRI was decreased by PEMF stimulation with the CNP[10].

The mechanisms by which electromagnetic fields can influence biological systems are not yet fully understood. An abundance of mechanisms have been proposed and a large number of them have been confirmed experimentally (for review see e.g. [24] or [25]). The most established mechanism is induction of an electrical potential due to changing magnetic flux density (Faraday's law). As a result ion motion (current) is altered, which in turn induces changes in synaptic potentials. Because synaptic potentials determine the likelihood of an action potential, this is a plausible mechanism for PEMF effects on neuronal activity: PEMF *per se* may not induce action potentials like TMS does, but it can change the temporal probability of action potentials. A recent paper found evidence for magnetic sensitivity in the low mT range of cryptochrome, a protein that is expressed throughout the tree of life including humans [26]. Human cryptochrome has indeed been shown to be sensitive to magnetic fields [27]. Cryptochrome is thus a candidate mediator of the analgesic [5] and antidepressant [6] effects of PEMF on humans. Mediation of PEMF effects by cryptochrome, being a protein sensitive to both light and magnetic fields, could also explain why PEMF effects were reported to be highly dependent on lighting conditions [28].

We found no differences between the effects induced by the two field strengths: apparently the intensities were equipotent. Dose-dependency of PEMF effects is generally very steep and has been described for different systems to occur below 1 mT [10], below 500 nT [29] and even below 50 nT [30]. It appears that the two field strengths used in our study (0.4 and 1.1 mT) both induced the maximum effect.

Concerning the penetration depth of our stimulation, it is often heard that TMS penetrates 1–2 cm, although H-coils can reach up to 6 cm [31]. Such statements are incomplete and inaccurate because what is implied is that TMS can induce action potentials at these depths. It is unknown whether magnetic fields have to induce action potentials in order to be effective at modulating biological functions. On the contrary, weak pulsed fields (PEMF) are effective in humans [5], [6] and it is highly unlikely that the direct induction of action potentials in the brain plays a role here. The magnetic permeability of biological tissues is very similar to that of air or vacuum meaning that the main factor determining the field strength of low frequency PEMF in the brain, apart from the current and the coil design, is the distance to the coil.

Thresholds for PEMF effects on living systems have been estimated at 500 nT [29] and even 50 nT [30]. Flux density was measured in our study at five distances from the coils with flux density values between 1 mT and 0.1 mT. These values fit very closely to an exponential dependence of flux density on distance. Extrapolating using this exponential dependence predicted flux densities of 500 and 50 nT to be reached at 2.2 and 2.9 cm from the coil respectively. This strengthens the notion that our stimulator induces biologically relevant magnetic fields in the brain [7].

In terms of safety, our newly designed magnetic stimulator conformed to the assessment criteria of the Dutch Work Group for the Classification of Instruments in University Hospitals (Wibaz) and is a class I, type B device according to the IEC 601-1988 norm. This indicates that the device is electrically safe to be used on humans. With regard to neurological safety, epileptic seizures are the main serious adverse event that can potentially be induced by magnetic stimulation. However, the risk of inducing seizures is controllable because it is a function of frequency and field strength[32]. Importantly, the stimulator described here falls well below the field strength described in the above paper: our field strength was not 100–220% [32] of the motor evoked potential threshold, but in the order of 0.05%. Therefore it seems highly unlikely that induction of epileptic seizures is a risk with the current setup. None of the volunteers could detect the stimulation or had adverse effects or other complaints so that the PEMF procedure appears to be completely safe.

A strength of this study is that we provide data from an actual measurement of the magnetic field whereas this is frequently omitted in PEMF reports. Additionally, we confirmed our prediction that HPT would increase after PEMF treatment and we measured many additional parameters. This study was sham-controlled and volunteers detected no difference so it was truly double-blind for the whole duration of the experiment. Although the treatment groups (PEMF-sham and sham-PEMF) were not fully balanced with respect to age, gender and handedness, the use of a crossover design precluded confusing group effects for treatment effects. In the paired design every subject served as their own control thus reducing the obscuring effects of intersubject variability.

As a limitation, the generalizability of this trial is limited because it was performed in a relatively small group (n = 20) consisting mostly of young women. Another limitation is that although the device permits considerable anatomical specificity of the PEMF stimulation, for this pilot we stimulated all locations simultaneously. Future studies should aim to elucidate the relative contribution of the individual electromagnets.

In summary, we built a magnetic stimulator capable of producing fluctuating magnetic fields with arbitrary temporal patterns within the 0–300 Hz frequency range. The use of an established coordinate system allows studies with anatomical specificity and integration with existing (EEG) literature. The use of nineteen small electromagnets makes it possible to stimulate specific neuroanatomical targets with the aim of modulating their function. This setup allows double-blind, sham-controlled experiments with arbitrary wave shape magnetic stimulation. These advantages, in addition to low cost and high safety, make this technology widely applicable for functional and clinical studies of the brain. As expected PEMF stimulation of the brain with this device caused increased pain tolerance in healthy subjects. At the same time, sensitivity to non-noxious thermal stimuli remained unchanged. We found no evidence for changes in emotional state and motor parameters that correlate with dopaminergic tone, thus it is unlikely that these would have mediated the changes in pain sensation.

## Supporting Information

Figure S1 **Electrical wiring diagram for the DC coupled amplifier with power supply (top) and amplifier (bottom).**
(TIF)
