# Supplementary figures and images for: A Novel Magnetic Stimulator Increases Experimental Pain Tolerance in Healthy Volunteers - A Double-Blind Sham-Controlled Crossover Study
Source: PLoS One. 2013 Apr 19;8(4):e61926. doi: 10.1371/journal.pone.0061926 (PMC3631254; doi:10.1371/journal.pone.0061926)

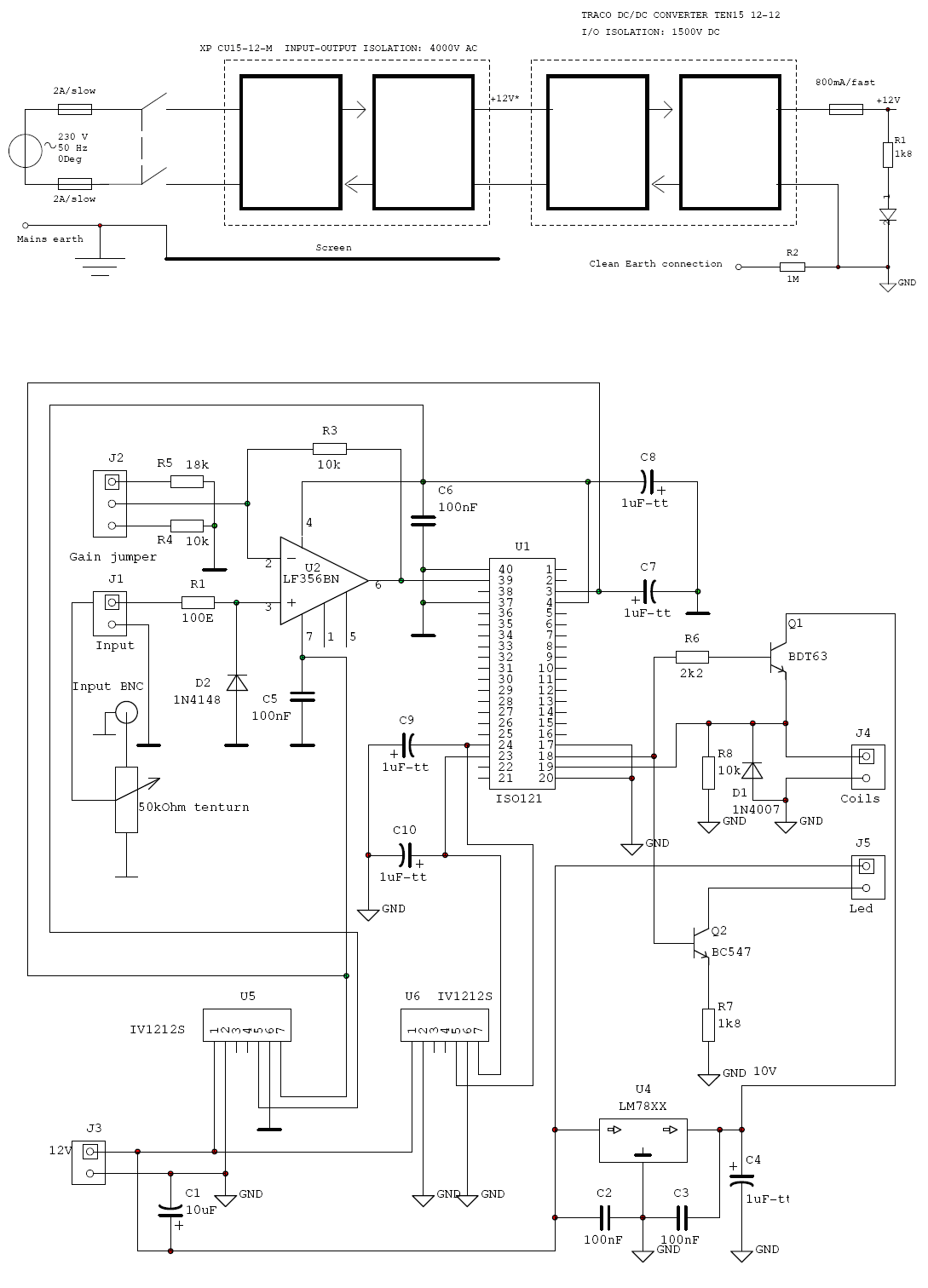

Supplement: Figure S1 — Electrical wiring diagram for the DC coupled amplifier with power supply (top) and amplifier (bottom). (TIF) [file pone.0061926.s001.tif]
